# Emergency surgical intervention for bulbar-cervical spinal cord hemorrhage: a case report and review of management strategies

**DOI:** 10.3389/fsurg.2025.1622953

**Published:** 2025-08-14

**Authors:** Chuan He, Qi Zhong, Ying Yang, Gang Cao

**Affiliations:** ^1^ Department of Neurosurgery, Zhuhai Hospital of Integrated Traditional Chinese and Western Medicine (Zhuhai Hospital Affiliated to Faculty of Chinese Medicine, Macau University of Science and Technology) Zhuhai City, China; ^2^ Zhuhai Integrated Traditional Chinese and Western Medicine Hospital Gongbei Community Health Service Center, Zhuhai City, China

**Keywords:** spinal cord hemorrhage, medullary and cervical spinal cord hemorrhage, emergency surgery, expansive open-door laminectomy, pedicle screw internal fixation

## Abstract

**Background and importance:**

Spinal cord hemorrhage (SCH), particularly involving the bulbar-cervical segment (medulla oblongata to C7), is a rare and life-threatening neurological emergency. Due to its anatomical proximity to respiratory, motor, and sensory centers, it often leads to catastrophic neurological deficits. Etiologies include vascular malformations, coagulopathy, or idiopathic causes, yet its low incidence (<1%) poses significant challenges in early diagnosis and management. Current evidence highlights emergency hematoma evacuation, adequate decompression, and spinal stabilization as critical for improving prognosis, though clinical validation through case-based data remains limited.

**Case presentation:**

A 28-year-old male presented with acute dyspnea, limb numbness, and progressive weakness (left 1/5, right 2/5 on the Medical Research Council scale) over 2 h. Neurological examination revealed a sensory deficit below the T4 dermatome and bilateral pyramidal signs. Imaging confirmed a hematoma extending from the medulla oblongata to C7, with no evidence of vascular malformations or trauma. Emergency microscopic hematoma evacuation (8 ml) was performed, combined with posterior cervical double-door laminectomy (C3–C7) and pre-contoured rod pedicle screw fixation. Postoperatively, mechanical ventilation was discontinued within 24 h. Sensory levels regressed to T8, and motor function improved progressively (left 3+/5, right 5/5 at 2-month follow-up). Postoperative imaging confirmed complete hematoma resolution and stable instrumentation.

**Conclusion:**

Bulbar-cervical SCH necessitates vigilant monitoring for cardiorespiratory compromise. Multidisciplinary emergency intervention—hematoma evacuation with decompression—effectively halts neurological deterioration. The dual-door laminectomy technique optimizes spinal canal expansion while preserving stability, providing a biomechanical foundation for neural recovery. This case underscores the pivotal role of early surgical decompression and stabilization in achieving favorable long-term outcomes for high-level SCH.

## 1 Background and importance

Spinal cord hemorrhage (hematomyelia), particularly involving the bulbar-cervical segment (medulla oblongata to C7), is an exceedingly rare neurological emergency associated with catastrophic neurological deficits due to its proximity to vital respiratory and motor centers. Early recognition and multidisciplinary intervention are critical for mitigating irreversible damage. We report a case of spontaneous bulbar-cervical hematomyelia, emphasizing the pivotal role of emergency surgical decompression and spinal stabilization in reversing neurological deterioration.

## 2 Case presentation

### 2.1 Patient information

On December 25, 2024, a 28-year-old male presented to the emergency department with acute-onset dyspnea, bilateral limb numbness, and progressive weakness [left 2/5, right 3/5 on the Medical Research Council (MRC) scale] over 2 h. Physical examination revealed hypoesthesia below the T4 dermatome, generalized hypotonia, and positive bilateral pyramidal signs.

### 2.2 Imaging findings

#### 2.2.1 Cranial CT

**Figure 1:**
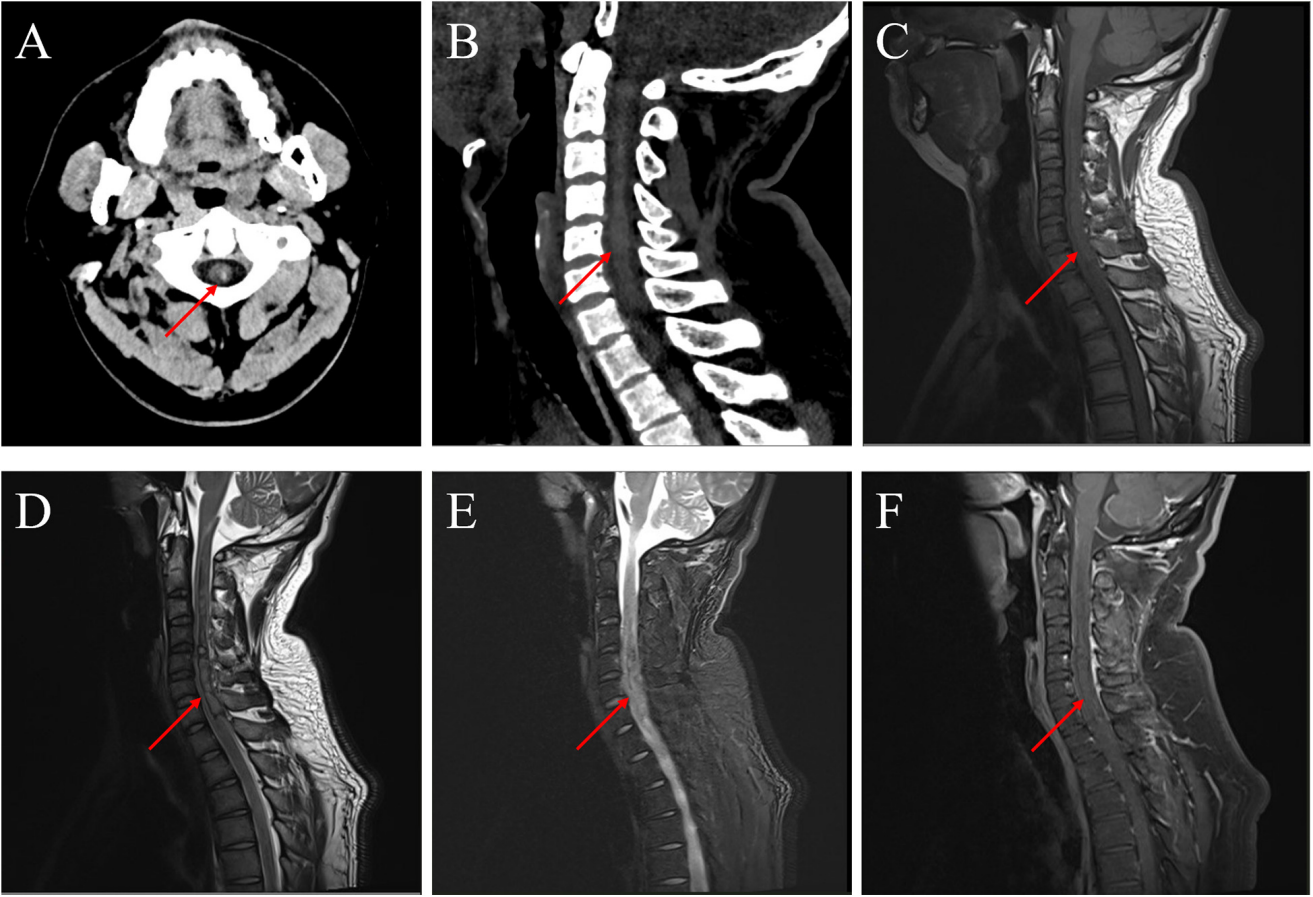
Preoperative imaging. **(A)** Emergency cranial CT showing medullary hemorrhage. **(B)** Sagittal view of cervical CT demonstrating the extent of hemorrhage. **(C**–**F)** Emergency MRI confirming hemorrhage in the medulla and cervical spinal cord.

A punctate hyperdense lesion within the medulla oblongata (Figure 1A).

#### 2.2.2 Cervical CT

Hyperdense intramedullary lesions extending from the medulla oblongata to C7, with significant cord swelling (Figure 1B).

#### 2.2.3 Cervical MRI

T1-isointense and T2-heterogeneous signals within the affected segments, accompanied by cord edema on fat-suppressed sequences. No contrast-enhanced lesions were observed (Figures 1C–F).

### 2.4 Treatment process

**Figure 2:**
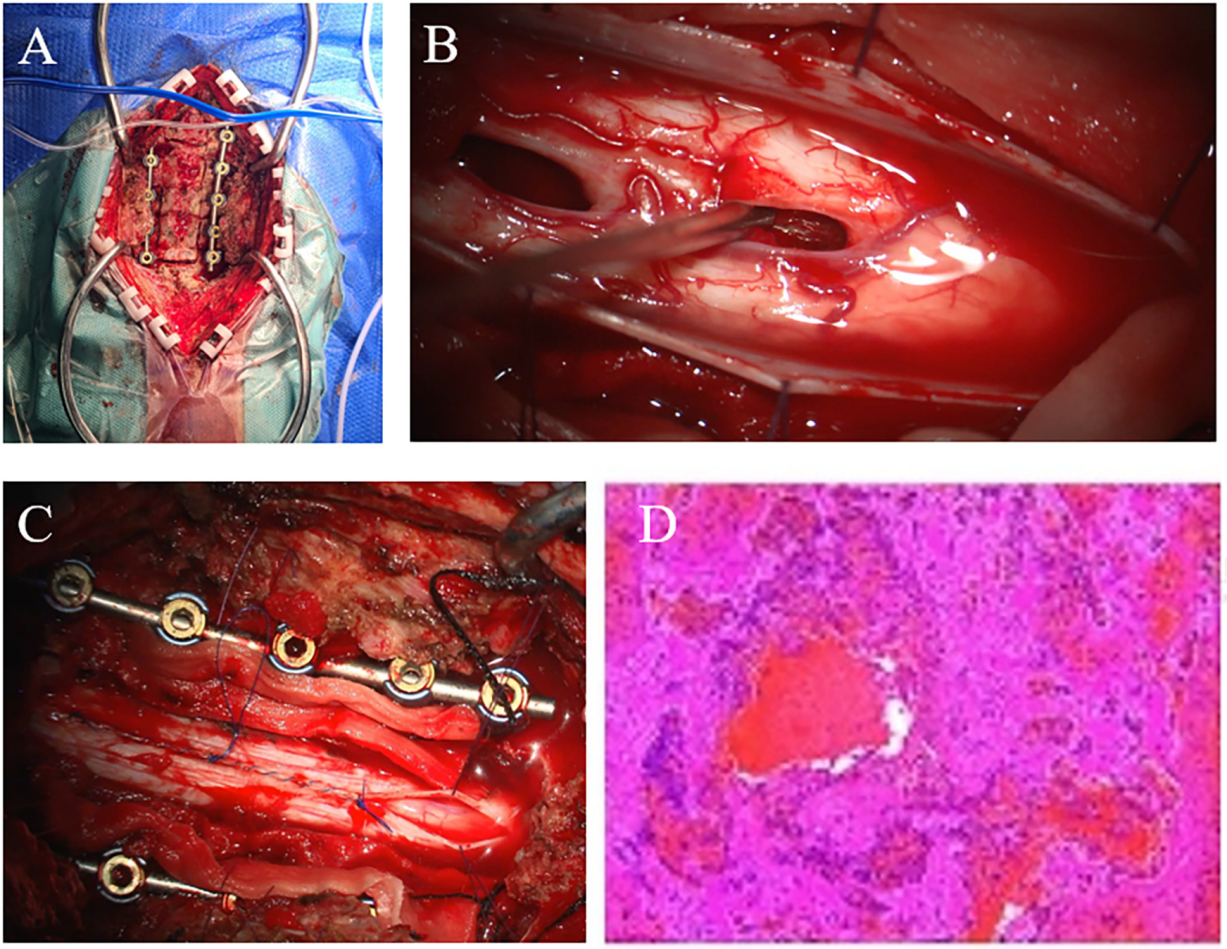
Intraoperative imaging and pathology. **(A)** Posterior cervical expansive open-door laminoplasty with screws inserted into the cervical lateral masses and pre-bent connecting rods, showing the extent of laminectomy. **(B)** Microscopic removal of intramedullary hematoma. **(C)** Suture the dura mater continuously and remove the lamina. **(D)** Pathological images.

**Figure 3:**
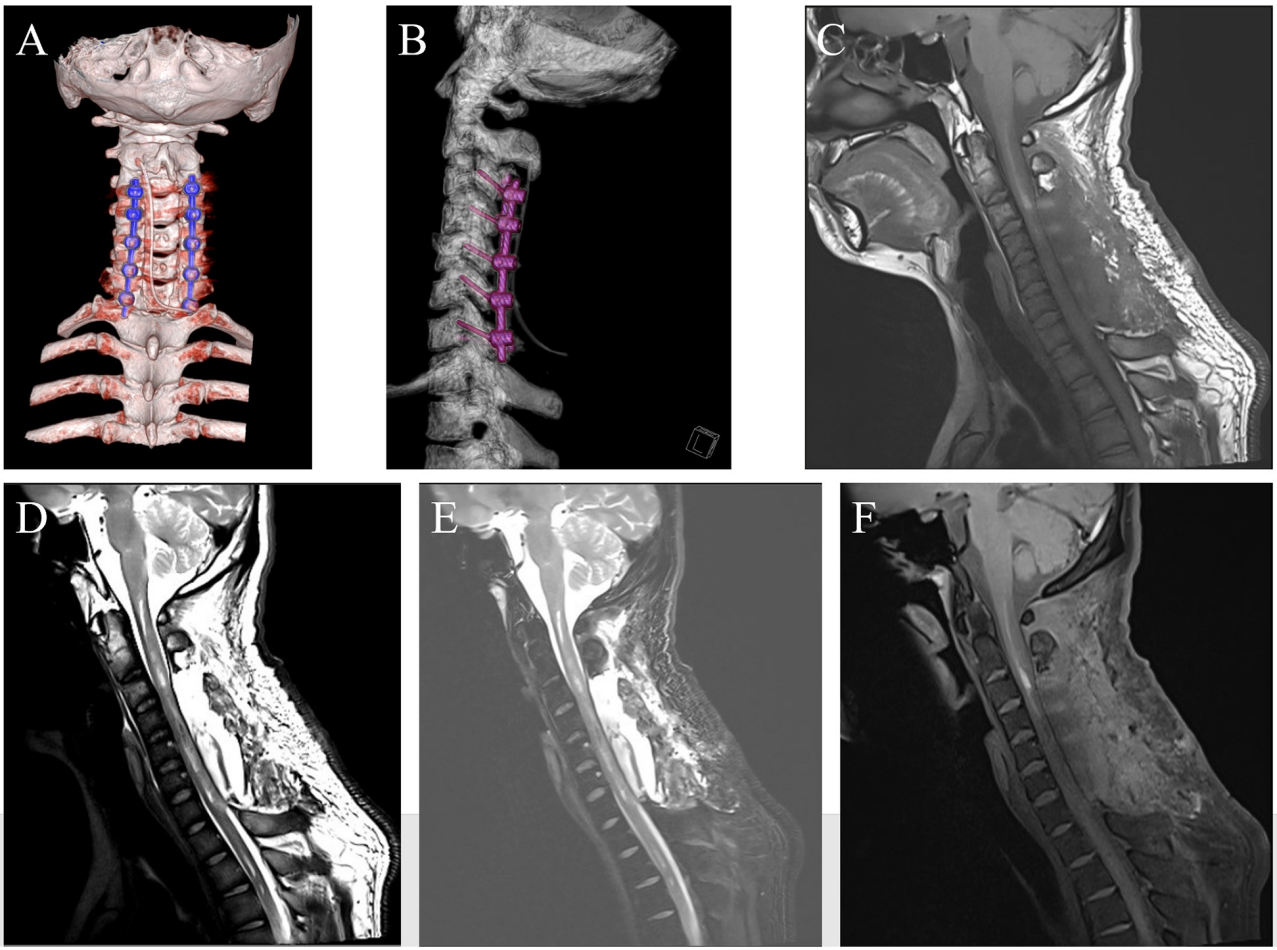
Postoperative follow-up imaging 1. **(A**,**B)** On postoperative day 1, cervical CT was reviewed. **(C**–**F)** At 1 week postoperatively, cervical MRI was reviewed.

After admission, the patient experienced progressive decline in muscle strength of both limbs, with grade 1 muscle strength on the left side and grade 2 on the right side. A multidisciplinary discussion was conducted involving neurosurgery, spine surgery, anesthesiology, radiology, and intensive care medicine to rule out contraindications for surgery. An emergency spinal angiography (DSA) was performed to exclude spinal arteriovenous malformation and dural arteriovenous fistula. Subsequently, the patient underwent intramedullary hematoma evacuation + posterior cervical laminectomy + pedicle screw internal fixation. During the surgery, the spinous process was resected, and the lower half of the third cervical lamina to the upper half of the seventh cervical lamina was removed. Screws and pre-bent connecting rods were inserted into the bilateral cervical lateral masses to serve as the basis for postoperative fixation. Then, under the microscope, the dura mater was longitudinally incised. The spinal cord was found to be swollen. A 2 mm incision was made in the posterior median sulcus to avoid vascular fistula, and the intramedullary hematoma was gently removed segment by segment, with a total volume of approximately 8 ml. Active bleeding was observed in the fourth to fifth cervical spinal cord segments. After electrocoagulation hemostasis, the surrounding pathological tissues and hematoma were sent for pathological examination, which reported thrombus formation (based on the subsequent pathological images, it was inferred that the bleeding was caused by cavernous angioma) (Figure 2). After hematoma evacuation, the tension of the spinal cord significantly decreased. The dura mater was repaired, and the muscles and skin were sutured to complete the surgery. After 24 h of postoperative respiratory support with a ventilator, sedation and analgesic treatments were gradually reduced. The patient's dyspnea was relieved, and the endotracheal tube was successfully removed. The patient's muscle strength did not continue to decline, and the sensory level recovered to the eighth thoracic nerve level. Follow-up cervical CT showed that the intramedullary hematoma was cleared, the swelling was reduced, and there was no new bleeding (Figure 3).

### 2.5 Follow-up and outcomes

**Figure 4:**
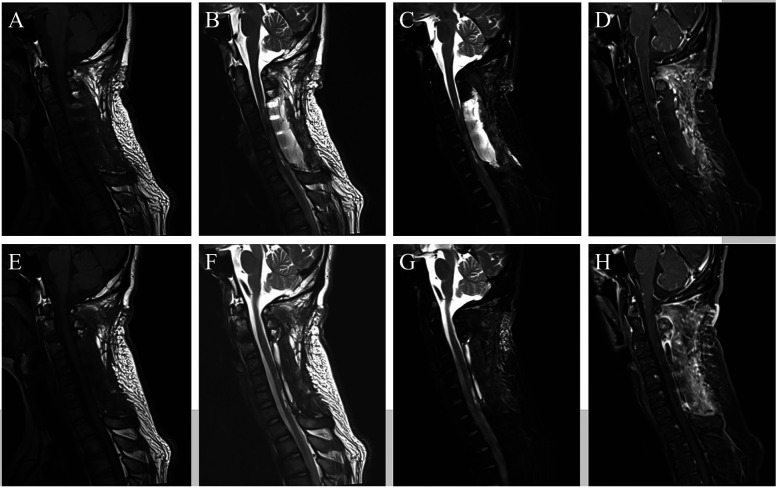
Postoperative follow-up imaging 2. **(A**–**D)** At 3 weeks postoperatively, cervical MRI was reviewed. **(E**–**H)** At 2 months postoperatively, cervical MRI was reviewed.

The patient's somatic sensation returned to normal, and muscle strength continued to improve. At 2 weeks postoperatively, the left limb had grade 3 muscle strength, and the right limb had grade 4. At 1 month postoperatively: the left limb had grade 3 muscle strength, and the right limb had grade 4+. At 2 months postoperatively: the left limb had grade 3+ muscle strength, and the right limb had grade 5. Follow-up cranial MRI examinations performed at 1 week postoperatively (Figure 3), 3 weeks postoperatively, and 2 months postoperatively (Figure 4) indicated that the spinal cord hemorrhage was completely cleared without recurrence and that the pedicle screws and connecting rods were securely fixed without deformation.

### 2.6 Informed consent

This study was approved by the Institutional Review Board of Zhuhai Integrated Traditional Chinese and Western Medicine Hospital. Written informed consent was obtained from the patient and his next of kin.

## 3 Discussion

### 3.1 Rarity of spinal cord hemorrhage and clinical characteristics of bulbar-cervical involvement

Spontaneous spinal cord hemorrhage (hematomyelia) is an exceedingly rare entity, accounting for less than 1% of all spinal cord pathologies, with bulbar-cervical involvement (medulla oblongata to C7) representing an even rarer subset (1). The catastrophic neurological deficits observed in such cases—exemplified by acute respiratory failure, limb paralysis, and sensory loss in our patient—stem from the anatomical vulnerability of the bulbar respiratory center and cervical motor/sensory pathways (2). Etiologically, idiopathic hematomyelia constitutes approximately 30% of cases, followed by vascular malformations, coagulopathy, or trauma (3). In this case, the absence of anticoagulant use, trauma, or vascular anomalies on DSA supported a diagnosis of idiopathic hemorrhage. The rarity of bulbar-cervical hematomyelia may be attributed to its intricate vascular anatomy, limited compensatory mechanisms, and the critical functional density of this region, necessitating heightened clinical vigilance and immediate intervention to prevent irreversible deficits (4).

### 3.2 Critical role of emergency hematoma evacuation and decompression in long-term outcomes

Neurological deterioration in spinal cord hemorrhage is driven by hematoma-induced mass effect, secondary ischemia, and inflammatory cascades. Current evidence underscores that surgical decompression within <12 h of symptom onset significantly improves neurological recovery (5, 6). Our patient underwent urgent multidisciplinary evaluation, including exclusion of vascular malformations via DSA, followed by microscopic hematoma evacuation within 6 h of admission. The surgical strategy—midline myelotomy with segmental hematoma removal (8 ml) and targeted hemostasis—aligns with the “early and radical decompression” principle (7), effectively reducing cord tension and mitigating secondary injury. Postoperative motor improvement (left: 1/5 → 3+/5; right: 2/5 → 5/5 on the MRC scale) and sensory level regression (T4 → T8) validate the necessity of timely intervention. Furthermore, multidisciplinary collaboration (neurosurgery, spine surgery, and critical care) proved indispensable for perioperative respiratory support and complication prevention, consistent with established protocols (8, 9).

### 3.3 Biomechanical advantages of posterior cervical double-door laminectomy with pre-contoured pedicle screw-Rod fixation

Spinal cord hemorrhage is often accompanied by spinal cord swelling and relative insufficiency of the spinal canal volume. Traditional laminectomy may lead to postoperative cervical instability. In this case, expansive open-door laminectomy was employed (9). While achieving adequate decompression, screws were pre-fixed to the bilateral cervical lateral masses. This method not only realized sufficient decompression within the spinal canal (from the lower part of the C3 layer to the upper part of the C7 layer) but also maintained the stability of the cervical spine. The use of pre-bent connecting rods during the surgery further conformed to the physiological curvature of the cervical spine, reducing the risk of stress concentration associated with internal fixation (10, 11). Postoperative imaging follow-up showed no displacement of the internal fixation and regression of spinal cord swelling, confirming the balanced advantages of this surgical technique in terms of decompression and stability. In line with the literature reports, this type of surgery can reduce the risk of postoperative cervical kyphosis and adjacent segment degeneration, providing a structural basis for long-term functional recovery of the patient (such as complete recovery of somatic sensation and continuous improvement of muscle strength) (12, 13).

## 4 Conclusion

This case of bulbar-cervical spinal cord hemorrhage (SCH) underscores three critical lessons for managing rare high-level SCH: (1) Early recognition of respiratory compromise and progressive neurological deficits is pivotal; (2) Multidisciplinary emergency intervention—combining hematoma evacuation, radical decompression, and spinal stabilization—serves as the cornerstone for improving prognosis; and (3) Biomechanical stability achieved through tailored techniques (e.g., double-door laminectomy with pre-contoured pedicle screw-rod fixation) is essential for long-term functional recovery. Future studies should prioritize elucidating the molecular mechanisms underlying idiopathic SCH and establishing evidence-based protocols for surgical timing and approach selection.

## Data Availability

The original contributions presented in the study are included in the article/Supplementary Material, further inquiries can be directed to the corresponding author.
